# Toxin Variability Estimations of 68 *Alexandrium ostenfeldii* (Dinophyceae) Strains from The Netherlands Reveal a Novel Abundant Gymnodimine

**DOI:** 10.3390/microorganisms5020029

**Published:** 2017-05-26

**Authors:** Helge Martens, Urban Tillmann, Kirsi Harju, Carmela Dell’Aversano, Luciana Tartaglione, Bernd Krock

**Affiliations:** 1 Alfred Wegener Institute Helmholtz Centre for Polar and Marine Research, Am Handelshafen 12, Bremerhaven 27570, Germany; helge.martens@outlook.de (H.M.); urban.tillmann@awi.de (U.T.); 2 Finnish Institute for Verification of the Chemical Weapons Convention (VERIFIN), Department of Chemistry, University of Helsinki, P. O. Box 55, Helsinki FI-00014, Finland; kirsi.harju@helsinki.fi; 3 Department of Pharmacy, University of Napoli Federico II, Via D. Montesano 49, Napoli 80131, Italy; dellaver@unina.it (C.D.); luciana.tartaglione@unina.it (L.T.)

**Keywords:** *Alexandrium ostenfeldii*, PSP-toxins, spiroimines, spirolides, gymnodimines, liquid chromatography-tandem mass spectrometry

## Abstract

*Alexandrium ostenfeldii* is a toxic dinoflagellate that has recently bloomed in Ouwerkerkse Kreek, The Netherlands, and which is able to cause a serious threat to shellfish consumers and aquacultures. We used a large set of 68 strains to the aim of fully characterizing the toxin profiles of the Dutch *A. ostenfeldii* in consideration of recent reports of novel toxins. *Alexandrium ostenfeldii* is known as a causative species of paralytic shellfish poisoning, and consistently in the Dutch population we determined the presence of several paralytic shellfish toxins (PST) including saxitoxin (STX), GTX2/3 (gonyautoxins), B1 and C1/C2. We also examined the production of spiroimine toxins by the Dutch *A. ostenfeldii* strains. An extensive liquid chromatography-tandem mass spectrometry (LC-MS/MS) analysis revealed a high intraspecific variability of spirolides (SPX) and gymnodimines (GYM). Spirolides included 13-desMethyl-spirolide C generally as the major compound and several other mostly unknown SPX-like compounds that were detected and characterized. Besides spirolides, the presence of gymnodimine A and 12-Methyl-gymnodimine A was confirmed, together with two new gymnodimines. One of these was tentatively identified as an analogue of gymnodimine D and was the most abundant gymnodimine (calculated cell quota up to 274 pg cell^−1^, expressed as GYM A equivalents). Our multi-clonal approach adds new analogues to the increasing number of compounds in these toxin classes and revealed a high strain variability in cell quota and in toxin profile of toxic compounds within a single population.

## 1. Introduction

In recent years, harmful algal blooms (HAB) have occurred with an increasing frequency [1,2], posing a serious risk for human health mainly by production of potent toxins which accumulate throughout the food chain. On the other hand, already back in 1938, a species of the genus *Alexandrium* was believed to be responsible for shellfish poisoning in Belgium. The causative species *Pyrodinium phoneus* [3] was successively inferred to be *Alexandrium ostenfeldii* [4]. Compared to other *Alexandrium* species, *A. ostenfeldii,* currently assumed to be conspecific with *A. peruvianum* [5], is a barely studied species of the genus. It has a wide geographical distribution including temperate waters of Europe [6], the eastern coast of North America [7], the western coast of South America [8], the southern tip of South America [9], New Zealand [10], and the west coast of Greenland [11]. In the past decade, *A. ostenfeldii* gained increasing attention because dense coastal blooms of this species were reported, e.g. from South America [8], the Northern Baltic Sea [12], the estuaries of the US East Coast [13,14] and, more recently, in The Netherlands [15]. These blooms represent a major concern for public health protection as some *Alexandrium* spp. are a source of paralytic shellfish toxins (PST) responsible for a neurotoxic syndrome. Firstly [16], PST in *A. ostenfeldii* were detected in strains from the Danish Limfjord, a finding that was later confirmed for other strains from other locations such as the Baltic Sea [5] and Chilean fjords [17]. PSTs represent one of the most serious groups of microalgal toxins and mainly consist of saxitoxin (STX), neosaxitoxin (NEO), gonyautoxins (GTX), and their *N*-sulfocarbamoyl variants, the B- and C-toxins [18].

Although the genus *Alexandrium* most often has been associated with PST production—as about 10 of the approximately 30 *Alexandrium* species are PST sources [19]—some strains of *A. ostenfeldii* lack the ability to produce PST. *Alexandrium ostenfeldii* currently is mainly linked to the production of spirolides (SPX), fast-acting toxins initially discovered in digestive glands of shellfish [20] and then in Atlantic Canadian strains of *A. ostenfeldii* [7]. Toxicity of SPX strongly depends on the cycloimine group which constitutes the pharmacophore [21]. Whereas the first studies identified spirolides A, B, C and D as well as two C and D isomers and some derivatives (13-desMethyl-spirolide C and D), subsequent work indicated that the diversity of these groups of compounds produced by *A. ostenfeldii* was much larger (Table 1), with spirolide G and its 20-Methyl derivative reported from Norway [22] and 27-Hydroxy-13,19-didesMethyl-spirolide C and a few other analogues described from a Mediterranean strain of *A. ostenfeldii*, some of which are still uncharacterized [23,24]. Two new SPX showing a unique dispiroketal system, spirolide H and I, were also isolated from Atlantic Canadian samples [25]. A thorough analysis of 36 strains of *A. ostenfeldii* from Greenland revealed the presence of 12 different SPX analogues, 8 of which had not been identified so far. Moreover, SPX composition varied considerably among strains indicating both a high variability of SPX within *A. ostenfeldii* and a high intraspecific variability in toxin profile as well [11].

To complicate the entire scenario even more, *A. ostenfeldii* has recently been reported to be also a producer of gymnodimines (GYM), another group of toxins which share the pharmacophoric cycloimine moiety with SPX [26]. Gymnodimines and SPX are thus both members of the spiroimine group of toxins (including also pinnatoxins, prorocentrolides, pteriatoxins and spiro-prorocentrimine) collectively related to spiroimine shellfish poisoning (SSP) based on their negative effects on neuromuscular, sensory, digestive and respiratory systems [27,28,29]. Gymnodimines only had been reported from some species of the genus *Karenia* [30,31], until 12-Methyl-gymnodimine A (12-Me-GYM A) was identified in *A. ostenfeldii* collected in brackish water from the east coast of the United States together with PST and SPX [26]. Just a few years later, a second GYM, namely gymnodimine A (GYM A), was found together with 12-Me-GYM A in *A. ostenfeldii* from The Netherlands [32], where this species since 2012 has formed recurrent noxious blooms in the Scheldt estuary [15]. In addition to GYM, a first characterisation of the Dutch bloom population [32] using 20 clonal strains revealed that all clones produced PST and SPX as well, with a particularly high intraspecific variability in the cellular amounts of spiroimines. SPX included 13-desMethyl-spirolide C as major compound, but the presence of low levels of several other SPX-like compounds in all clones was noted [32] yet not further investigated.

As a follow-up of the study, the major aim of the present study was to fully characterize *A. ostenfeldii* from the Netherland by liquid chromatography-tandem mass spectrometry regarding these minor components of the spiroimine group of toxins. Moreover, we aimed to more thoroughly analyze the intraspecific variability of the toxin profile both in a qualitative and quantitative perspective, by using a large set of 68 clonal strains. During our study, a Finnish research group identified a new GYM, gymnodimine D, as the major GYM in Baltic *A. ostenfeldii* strains [33]. Moreover, this group identified more than 30 other albeit minor GYM-like compounds [33], indicating that a considerable diversity of GYM might be a common feature for *A. ostenfeldii*. These findings prompted us to include this new set of compounds in our toxin screening.

## 2. Materials and Methods

### 2.1. Culture

*Alexandrium ostenfeldii* used for this study were collected in July 2013 during a bloom in the Ouwerkerkse Kreek (51°62’ N, 3°99’ E), The Netherlands [32]. A total of 68 strains were established by single-cell isolation using microcapillary into individual wells of a 96-well plate pre-filled with diluted North Sea water with a salinity of 10. Culture medium was sterile-filtered (0.2 μm VacuCap filters, Pall Life Sciences, Dreieich, Germany) and enriched with 1/2 strength K-medium [35] that was modified by omitting the addition of ammonium. The pH of the culture medium was adjusted to pH 8.0 (EcoScan Series, Eutech instruments, Thermo Scientific, Dreieich, Germany) by adding 1 M hydrochloric acid. Stock cultures were routinely grown non-axenic at a salinity of 10, a temperature of 15 °C, and an incoming photon flux density of 50 μmol photons m^−2^ s^−1^ on a 16:8 h light–dark cycle.

### 2.2. Toxin Screening

For toxin analysis, all strains were grown in 70 mL plastic culture flasks. For each harvest, cell density was determined by settling Lugol-fixed samples (2% final concentration) and counting >600 cells in a 2 mL counting chamber with an inverted microscope (Zeiss Axiovert 40C, Göttingen, Germany) at 200× magnification. Cultures at a cell density ranging from 1000 to 3500 cells mL^−1^ were harvested by centrifugation (Eppendorf 5810R, Hamburg, Germany) at 3220× *g* for 10 min. Subsamples of 15 mL for analyses of spiroimine toxins and of 50 mL for analyses of PST were taken. Cell pellets were transferred to 1 mL microtubes, again centrifuged (16,000× *g*, 5 min, Centrifuge 5415R, Eppendorf, Hamburg, Germany), and stored frozen (−20 °C) until extraction.

### 2.3. Post-Column Derivatization Analyses of PST

Cell pellets for PST analysis were extracted with 0.03 M acetic acid by reciprocal shaking at maximum speed (6.5 m s^−1^) for 45 s in a FP 120 FastPrep instrument (Bio101, Thermo Savant, Illkirch, France). After centrifugation, the supernatant was spin-filtered (pore-size 0.45 mm, Millipore Ultrafree, Eschborn, Germany), the filtrate transferred into a HPLC vial (Agilent Technologies, Waldbronn, Germany) and kept at −20 °C until analysis. PST analyses were performed as ion pair chromatography on an octadecyl stationary phase (C18) with two subsequent isocratic elutions: 15 min with 6 mM 1-octanesulphonic acid and 6 mM 1-heptanesulphonic acid in 40 mM ammonium phosphate, adjusted to pH 7.0 with dilute phosphoric acid and 0.75% tetrahydrofuran (THF) and then switched within 1 min to 13 mM 1-octanesulphonic acid in 50 mM phosphoric acid adjusted to pH 6.9 with ammonium hydroxide, 15% of acetonitrile and 1.5% of THF for 24 min. Post-column derivatization was performed with 10 mM periodic acid in 550 mM ammonium hydroxide and subsequently 0.75 N nitric acid: both reagents were added at a constant flow of 4 mL min^−1^. Toxin derivatives were detected by fluorescence detection (λ_ex_ = 333 nm; λ_em_ = 395 nm). All toxins were identified and quantitated against an external calibration curve containing C1/2, B1, STX, NEO, GTX-1 to 4, dcGTX-2/3 and dcSTX. These toxins were purchased from the certified reference material (CRM) program of the National Research Council (NRC, Halifax, NS, Canada). Limits of detection (LOD) were defined as the signal-to-noise (S/N) ratio higher than three and are given on a per-cell basis. However, LODs were calculated with the mean cell numbers of all strains and thus only reflect orders of magnitude rather than exact values (Appendix A, Table A1).

### 2.4. Extraction

Cell pellets were transferred to 2 mL microcentrifuge tubes (neoLab, Heidelberg, Germany) containing 0.5 g lysing matrix D (Thermo Savant, Illkirch, France). Subsequently, the pellets were suspended in 500 µL methanol (Merck, Darmstadt, Germany) and homogenized by reciprocal shaking at maximum speed (6.5 m s^−1^) for 45 s in a FP 120 FastPrep instrument (Bio101, Thermo Savant, Illkirch, France). After homogenization, the samples were centrifuged (16,000 g, 15 min, 4 °C, Centrifuge 5415R, Eppendorf, Hamburg, Germany) and the supernatant was transferred to a spin-filter (pore-size 0.45 mm, Millipore Ultrafree, Eschborn, Germany) and centrifuged for 30 s at 3220 g. Filtrates were transferred into HPLC vials (Agilent Technologies, Waldbronn, Germany) and stored at −20 °C.

### 2.5. Analyses of Spiroimines by Liquid Chromatography-Tandem Mass Spectrometry (LC-MS/MS)

The spiroimine measurements were performed on a triple-quadrupole mass spectrometer (API 4000 Q Trap, AB-Sciex, Darmstadt, Germany) with a Turbo V ion source coupled to an Agilent 1100 LC liquid chromatograph (Waldbronn, Germany). The LC was equipped with a solvent reservoir, in-line degasser (G1379A), binary pump (G1311A), refrigerated autosampler (G1329A/G1330B) and a temperature-controlled column oven (G1316A). The separation was carried out on an analytical C8 reverse phase column (50 mm × 2 mm) packed with 3 μm Hypersil BDS 120 Å (Phenomenex, Aschaffenburg, Germany) and thermostated at 20 °C. The flow-rate was 0.2 mL min^−1^ and a gradient elution performed, where eluent A consisted of water and eluent B was methanol/water (95:5 v/v), both containing 2.0 mM ammonium formate and 50 mM formic acid. Initial conditions were 5% of eluent B. After injection, a linear gradient to 100% B in 10 min was performed and followed by isocratic elution until 20 min. Then the eluent composition was set to initial conditions within 1 min followed by 9 min column equilibration. The total run time was 30 min. The mass spectrometric parameters were as follows: Curtain gas: 20 psi, CAD (collision activated dissociation) gas: medium, ion-spray voltage: 5500 V, temperature: 650 °C, nebulizer gas: 40 psi, auxiliary gas: 70 psi, interface heater: on, declustering potential: 121 V, entrance potential: 10 V, exit potential: 22 V. The collision energy was 57 V for each transition.

For spiroimine screening [M + H]^+^ > [M + H – H_2_O]^+^ transitions of the known GYM were included into the SRM method of LC-MS analysis: *m/z* 508 > 490 for GYM A, *m/z* 522 > 504 for 12-Me-GYM A and *m/z* 524 > 506 for gymnodimines B, C and D. In addition, we included two transitions (*m/z* 510 > 492 and 526 > 508) of as yet unidentified GYM in Baltic strains of *A. ostenfeldii* [33]. Furthermore, transitions of all SPX reported in the literature were included in the method (Table 2).

Measurements were performed in the positive ion-mode and dwell times of 40 ms were used for each transition. SPX were calibrated against an external calibration curve of 13-desMethyl-spirolide C (certified reference material; NRC, Halifax, NS, Canada) and expressed as 13-desMethyl-spirolide C equivalents. For the calibration curve, the following concentrations of 13-desMethyl-spirolide C were used: 10 pg μL^−1^, 50 pg μL^−1^, 100 pg μL^−1^ and 1000 pg μL^−1^. Likewise, GYM were calibrated against an external calibration curve of GYM A (CRM; NRC, Halifax, NS, Canada) and expressed as GYM A equivalent. 12-Methyl-gymnodimine A was purchased from Biomol GmbH (Hamburg, Germany) and used for compound identification. For quantifying GYM, the following concentrations of a standard solution of GYM A were used: 10 pg μL^−1^, 50 pg μL^−1^, 500 pg μL^−1^ and 1000 pg μL^−1^. Limits of detection were defined as S/N ≥ 3 and are given as means of all strains in Appendix A, Table A2. Data acquisition and processing was performed with the Analyst Software (version 1.5, AB Sciex, Darmstadt, Germany). In addition, collision-induced dissociation (CID) spectra were recorded of all detected compounds.

### 2.6. Analyses of Spiroimines by Liquid Chromatography–High-Resolution Mass Spectrometry (LC–HRMS)

#### Compound (**6**) by Orbitrap Fusion

Strain OKNL20 was measured with Orbitrap Fusion high-resolution mass spectrometer (Thermo Scientific, San José, CA, USA) connected to Dionex Ultimate 3000 UHPLC (Thermo Scientific, Dionex, Germering, Germany). Heated ESI source (HESI) was used with a positive mode ionisation. EASY-IC^TM^ ion source with fluoranthene was used for internal calibration. The separation was performed with a C18 column (Acquity UPLC BEH 2.1 × 50 mm, 1.7 µm, Waters). The elution gradient was started with 90% of eluent A (0.1% HCOOH in MilliQ water) and 10% of eluent B (0.1% HCOOH in acetonitrile). The elution gradient was changed from eluent A 90% to A 10% (0–10 min), changed back to eluent A 90% at 10.1 min, and equilibrated until 12 min with eluent A 90%. The flow rate was 0.6 mL min^−1^, the column oven temperature was set to 40 °C, and the injection volume was 3 µL. The scan range was 100–600 *m/z*, resolution: 120,000, spray voltage: 3 kV, ion transfer tube temperature: 350 °C, and vaporizer temperature: 300 °C. The product ions of MS^2^ fragmentation at *m/z* 510 are listed in Table 3. The results were obtained with collision-induced dissociation (CID) energy of 35%. Higher-energy collisional dissociation (HCD) energy of 35% was also applied for fragmentation, and it produced the product ion typical for GYM (*m/z* 136). The mass tolerances were set to 3.00 ppm, and the charge was +1.

### 2.7. Accurate Mass Measurements of Spiroimines by Hybrid Linear Ion Trap Orbitrap FTMS

#### 2.7.1. Solid Phase Extraction (SPE) Clean-Up

Combined extracts of the *A. ostenfeldii* strains OKNL35 and OKNL43 were dissolved in 250 µL of H_2_O and loaded on a Strata-X C18, 6 mL (Phenomenex, Torrance, CA, USA) equilibrated with H_2_O. The cartridge was washed with 10 mL of H_2_O and then eluted with 10 mL of H_2_O/CH_3_CN (7:3, v/v), 10 mL of H_2_O/CH_3_CN (1:1, v/v), 10 mL of acetonitrile and 10 mL of MeOH. Each SPE eluate was evaporated to dryness and dissolved in 1.5 mL of MeOH before analyses. 

#### 2.7.2. Liquid Chromatography–High-Resolution Mass Spectrometry (LC–HRMS)

The analyses were performed on the crude extract of *A. ostenfeldii* strains OKNL35 and OKNL43 and SPE eluates by using a hybrid linear ion trap LTQ Orbitrap XL™ Fourier transform mass spectrometer (FTMS) equipped with an ESI ION MAX™ source (Thermo Fisher, San José, CA, USA) coupled to a Dionex Ultimate 3000 system which included a solvent reservoir, in-line degasser, quaternary pump and refrigerated autosampler and column oven. The following conditions were used: a 3 µm Hypersil C8 BDS, 50 × 2.00 mm column (Phenomenex, Torrance, CA, USA) at room temperature. Eluent A was H_2_O and B was a 95% acetonitrile/H_2_O solution, both containing 2 mM ammonium formate and 50 mM formic acid as suggested [40]. The flow rate was 0.2 mL min^−1^. A fast-gradient elution, 10–100% B in 10 min followed by 100% B for 15 min, was used in most of the experiments. A slow-gradient (10–30% B over 2 min, 30–80% B over 16 min, 80–100% B in 3 min, and hold 5 min) was also used to separate potentially interfering compounds. Injection volume was 5 μL. Full scan high-resolution mass spectrometry (HRMS) experiments (positive ions) were acquired in the *m/z* 400–1000 range at a resolving power (RP) of 100,000 (FWHM at *m/z* 400). The following source settings were used: spray voltage = 4.2 kV (SPX) and 4.5 kV (GYM), capillary temperature = 400 °C, capillary voltage: 14 V (SPX) and 20 V (GYM), sheath gas flow = 27 (SPX) and 35 (GYM), auxiliary gas flow = 0 (SPX) and 5 (GYM), and tube lens voltage = 100 V. Calculation of elemental formulae was performed on the mono-isotopic peak of each ion cluster using Xcalibur software v2.0.7 (Thermo Fisher, San José, CA, USA) at a 5 ppm mass tolerance.

## 3. Results

### 3.1. Toxin Profile

**Figure 1:**
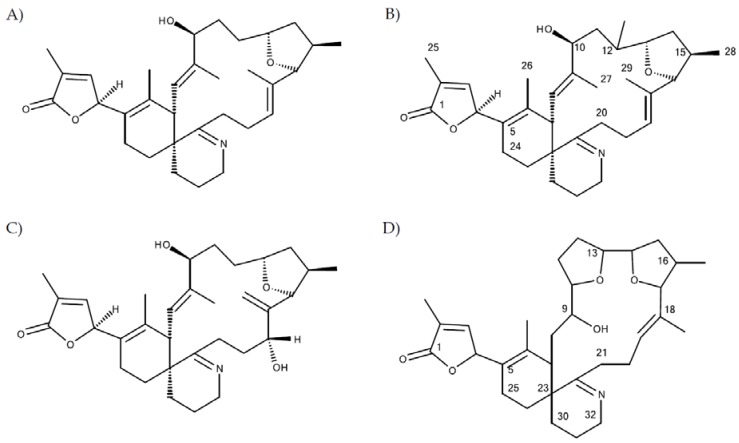
Structures of: (**A**) gymnodimine A; (**B**) 12-Methyl-gymnodimine A; (**C**) gymnodimine C and (**D**) gymnodimine D.

All 68 strains showed the same PST profile consisting of C1/C2, GTX2/3, B1 and STX with variable cell quotas (Table 4). Several compounds corresponding to various spiroimines were also detected (Table 2). Five spiroimines were detected in the strains by SRM screening and four of them were identified by comparison of retention times and CID spectra with those of previously isolated compounds. These four spiroimines include 13-desMethyl-spirolide C (Table 1), 27-Hydroxy-13-desMethyl-spirolide C, gymnodimine A and 12-Methyl-gymnodimine A (Figure 1). As the fifth spirolide, a yet unknown spirolide with a pseudo-molecular ion at *m/z* 694 (**1**) was detected. 

**Figure 2:**
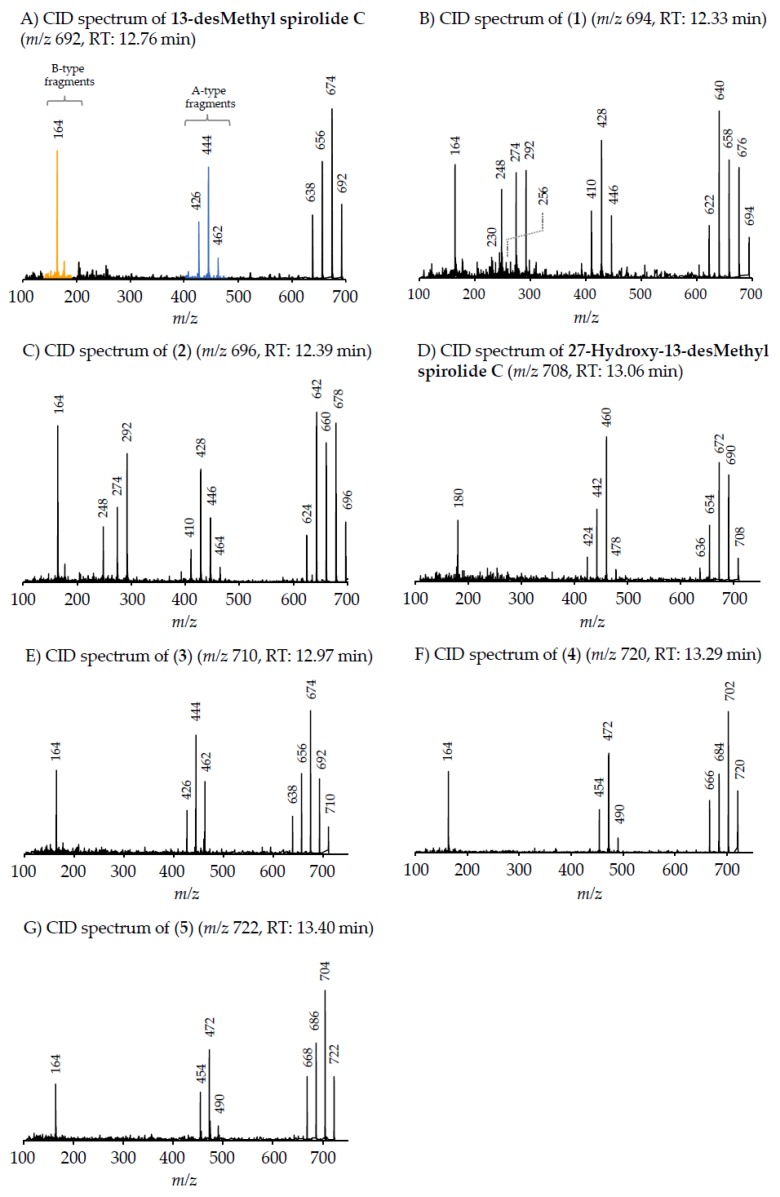
Collision-induced (CID) spectra of all determined spirolides: (**A**) 13-desMethyl-spirolide C; (**B**) compound (**1**); (**C**) compound (**2**); (**D**) 27-Hydroxy-13-desMethyl-spirolide C; (**E**) compound (**3**); (**F**) compound (**4**) and (**G**) compound (**5**).

**Figure 3:**
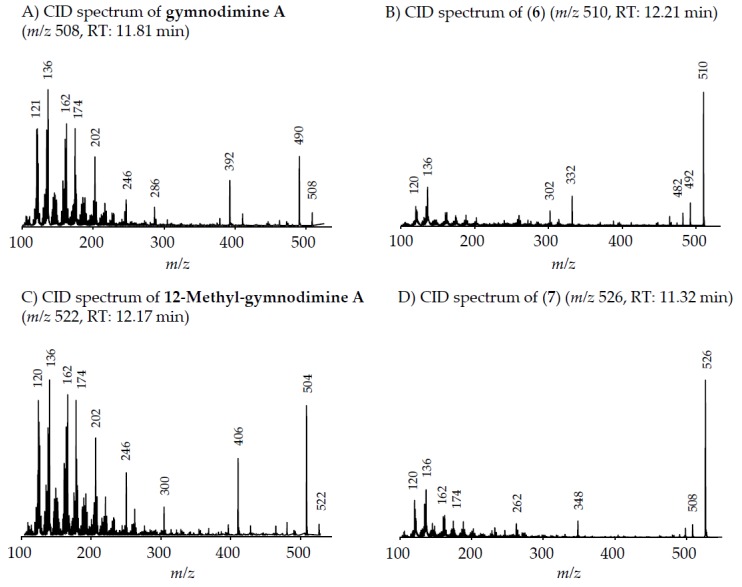
CID spectra of all determined gymnodimines: (**A**) gymnodimine A; (**B**) compound (**6**); (**C**) 12-Methyl-gymnodimine A and (**D**) compound (**7**).

Precursor ion scans of the characteristic spirolide fragment at *m/z* 164, which is characteristic for SPX [41], revealed the presence of four more precursor ions with *m/z* 696 (**2**), *m/z* 710 (**3**), *m/z* 720 (**4**) and *m/z* 722 (**5**). The CID experiments of these precursor masses displayed the typical A- and B-type fragments characteristic for SPX (Figure 2). In addition, high-resolution mass spectrometric (HRMS) measurements further confirmed the presence of the known spiroimines as well as of compounds (**2**)–(**5**) and additionally revealed the presence of two more gymnodimines with *m/z* 510 (**6**) and *m/z* 526 (**7**) (Table 5). Finally, HRMS measurements and CID spectra were recorded for all the detected pseudo-molecular ions to obtain information on the identity of these compounds. The accurate mass of pseudo-molecular ion of (**1**) at *m/z* 694.4322 fitted with an elemental composition of C_41_H_60_O_8_N (Table 5). In contrast, CID spectra of compounds *m/z* 510 (**6**) and *m/z* 526 (**7**) showed typical GYM fragments (Figure 3) e.g., the loss of water and a product ion at *m/z* 136.

**Figure 4:**
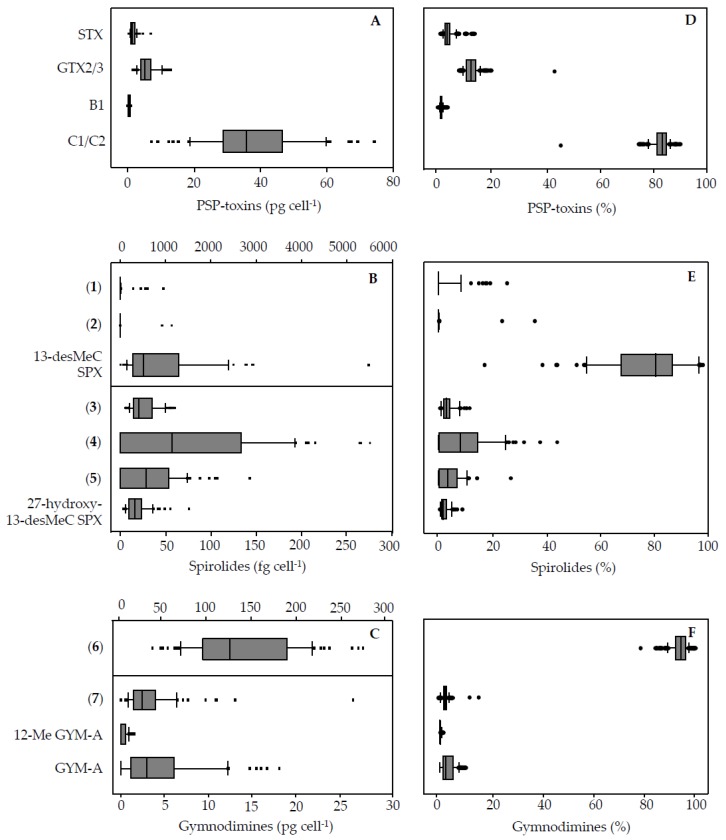
Box–Wisker Plots for absolute (**A**–**C**) and relative values (**D**–**F**) of PST, spirolides and gymnodimines.

The typical A-type SPX fragment cluster of (**1**) (Figure 2B) was downshifted of 16 Da in comparison to 13-desMethyl-spirolide C [41,42]. In addition to the B-type fragment at *m/z* 164, ions not commonly observed in SPX CID spectra appeared at *m/z* 248, 274 and 292. The CID spectrum of (**2**) (Figure 2C) contained the same type-A and -B fragments as (**1**), while its pseudo-molecular ion and associated water losses were up-shifted of 2 Da. Compound (**3**) displayed an identical CID spectrum as 13-desMethyl-spirolide C except for the pseudo molecular ion cluster, which was up-shifted of 18 Da (Figure 2E). In contrast, the C1 to C11 part (Figure 4) of (**4**) (Figure 2F) was identical to that of 13-desMethyl-spirolide C (Figure 2A), as mass differences from the pseudo molecular ions and the A-type fragments of both compounds were identical (230 Da; *m/z* 692–462 and 720–490). The mass difference between (**4**) and 13-desMethyl-spirolide C was 28 Da. Compound (**5)** in turn showed an identical CID spectrum as (**3**) except for an up-shift of 2 Da of the pseudo molecular ion cluster (Figure 2G).

### 3.2. Toxin Variability

PST cell quotas among strains ranged from 11.3 to 88.2 pg cell^−1^ (mean 45.7 pg cell^−1^) (Appendix A: Table A3, Figure 4A). The PST profile was very consistent among all the strains (Figure 4D) with a dominance of C1/C2 (mean relative abundances of 82.3%) and lesser contributions of GTX2/3 (12.8%), STX (3.8%), and B1 (1.1%). As the only exception, one strain (OKNL68, Appendix A, Table A3) had a slightly different relative composition and contained relatively less C1/C2 (44.6%) and higher relative contributions of the other compounds (GTX2/3: 42.1%, STX: 10.2%; B1: 3.1%).

Total SPX cell quotas (expressed as 13-desMethyl-spirolide C equivalents) among strains ranged from 0.09 to 5.6 pg cell^−1^ (mean: 1.2 pg cell^−1^) (Appendix A: Table A4, Figure 4). For all spirolides there was a high variability both in cell quota and relative contribution to total SPX among the strains (Figure 4B,E). One spirolide was detected in all strains (13-desMethyl-spirolide C), whereas other spirolides were below detection limit in several strains (Table 4). 13-desMethyl-spirolide C generally dominated the spirolide profile (Figure 4E) but nevertheless relative contribution among strains ranged from 17% to 98%. 27-Hydroxy-13-desMethyl-spirolide C was present in 37 strains and showed the lowest range of relative contribution to total SPX (0–8.8%). Compounds (**1**) and (**2**) were detected in just a few strains (7 and 2 strains, respectively), and relative contribution varied greatly. Likewise, cell quotas of individual spirolides varied among strains with fold changes between a minimum (defined here as the strains with the lowest amount above detection limit) ranging from 1-fold (compound (**2**)) to 273-fold (13-desMethyl-spirolide C) (Table 4).

The total amount of GYM (expressed as GYM A equivalents) per cell among strains ranged from 40.7 to 295.0 pg cell^−1^ (mean: 144.7 pg cell^−1^) (Appendix A Table A4). Overall, (**6**) was the dominant compound. It was detected in all the strains and accounted for 77% to 100% of all GYM content. As for the other gymnodimines, GYM A and (**7**) were not detected in about 10% of the strains, while 12-Me-GYM A was detected in about half of the strains (Table 4). Cell quotas of individual gymnodimines varied considerably among strains (Figure 4C,D) with fold changes between a minimum (defined here as the strain with the lowest amount above detection limit) ranging from 5-fold (12-Me-GYM A) to 59-fold (GYM A) (Table 4).

## 4. Discussion

Our study of multiple clones of a brackish water population of *A. ostenfeldii* revealed a conserved qualitative PST profile but high quantitative variability of individual PST, and a high structural diversity and quantitative variability of spiroimines. As we analysed intracellular compounds, and autonomous spiroimine production by bacteria has never been reported, we argue that a potential contribution of extracellular bacteria in the cultures is unlikely and that secondary metabolite variability indeed is a phenotypic trait of the different strains. Previously, relatively few spiroimines have been reported in the literature [43]. This is probably because this group of compounds normally does not receive as much attention as toxins that are known to cause illness in humans. Spiroimines indeed show little oral toxicity and thus are not regarded as a risk for consumers of contaminated shellfish. However, they do have severe and fast neurotoxic effect after intraperitoneal injection into mice and thus deserve attention. With this work, we add five novel spirolides and two new gymnodimines produced by *A. ostenfeldii* to the growing list of spiroimines. Furthermore, our results highlight a high variability of toxin cell quotas among multiple strains of one algal population. Toxin cell quotas are well known to be modulated by environmental factors [44,45]. In our study, however, all strains were grown under identical environmental conditions (growth medium, nutrients, light and temperature) and were sampled for toxin analysis during active growth at a comparable cell density of about 1000–3500 cells mL^−1^, which corresponds to a mid-exponential phase. Moreover, for a selected number of strains of the Dutch *A. ostenfeldii* population, detailed culture experiments [32] revealed a generally low deviation of replicate cultures (relative standard deviation of 10% for PST, 8% for GYM, and 7% for SPX). We therefore conclude that our non-replicated analysis of multiple strains indeed reflects considerable genetically based intraspecific variability in the quantity of produced toxins.

Variability of various traits is well known within microalgal populations [46,47,48]. Such high variability might facilitate widely variable phenotypic responses within a population to changes in biotic and/or abiotic conditions, and make populations resilient to changes in environmental and climatic conditions. Variability thus may be the result of adaption to variable environmental conditions [49], which presumably are high in the small and shallow Ouwerkerkse Kreek. Future studies on the Dutch bloom population and/or other toxic microalgal blooms are needed to determine if and to what extend phenotypic (e.g., chemical) variability determined here coincide with genotypic (e.g., as estimated with microsatellite markers) variability.

### 4.1. Paralytic Shellfish Poisoning Toxins

For PST, the toxin profile was identical for all strains with very little intraspecific variability in relative contribution. It presented a dominance of C1/2, lesser amounts of GTX2/3 and STX, and traces of B1. This agrees with earlier results for a small set of strains from the same *A. ostenfeldii* population [32]. Compared to other *A. ostenfeldii* strains, this PST profile is identical to that of *A. ostenfeldii* from the US coast, but different from that of strains from the Baltic Sea and Peru, which lack C1/2 and B1 [5], and different from a strain from China, which is unique in producing just neosaxitoxin [5].

In contrast to the PST profile, quantitative variability in cellular PST content was high with fold-changes between the lowest and highest quota ranging from 8 (GTX 2/3) to 18 (SPX) (Table 4). Total PST contents measured in the present study and based on analysis of 68 *A. ostenfeldii* strains was in the range 11.3–88.2 pg cell^−1^ (7.8-fold), in good agreement with the total toxin content reported earlier (9.5–51.0 pg cell^−1^; 5.4-fold) [32], although these authors included only 20 *A. ostenfeldii* strains in their study. This suggests that an analysis of a higher number of strains does not provide further insights into PST variability within a single population.

### 4.2. Spiroimine Structural Diversity

#### 4.2.1. Spirolides

Differently from PST, our study of a larger number of strains of the Dutch *A. ostenfeldii* population revealed interesting information about spiroimines. Whereas the presence of 13-desMethyl-spirolide C and 27-Hydroxy-13-desMethyl-spirolide C as dominant spirolides had been reported in the previous study [32], we detected and characterised a total of five additional and yet unreported spirolide analogues. This identification is based on the characteristic fragmentation pattern of SPX, which consists of three fragment groups: the first fragment cluster consists of the protonated molecular ion and several water losses, typically in the mass range between 650 and 750 Da. The second characteristic SPX fragment group (A type, Figure 2A) is produced by the loss of the butenolide side chain of the molecule, which is formed after a retro-Diels–Alder opening of the SPX macrocycle and subsequent water losses. This fragment cluster typically occurs in the mass range of 400 to 500 Da [41], except for G-type SPX, where this cluster is shifted to lower masses [22]. The third SPX fragment group (B-type, Figure 2A) consists of the cyclic imine ring with an ethylene rest of the macrocycle at *m/z* 150, 164 or 180 (depending on the degree of methylation and/or hydroxylation). In addition to the two known SPX, the five new SPX with pseudo molecular ions at *m/z* 694, 696, 710, 720 and 722 could be characterized by their typical fragmentation pattern contained in CID spectra (Figure 2A–G). Although structure elucidation ultimately will require NMR analysis, the recorded CID spectra nevertheless allow a clear mass spectrometric characterization.

##### Compound **1**

**Figure 5:**
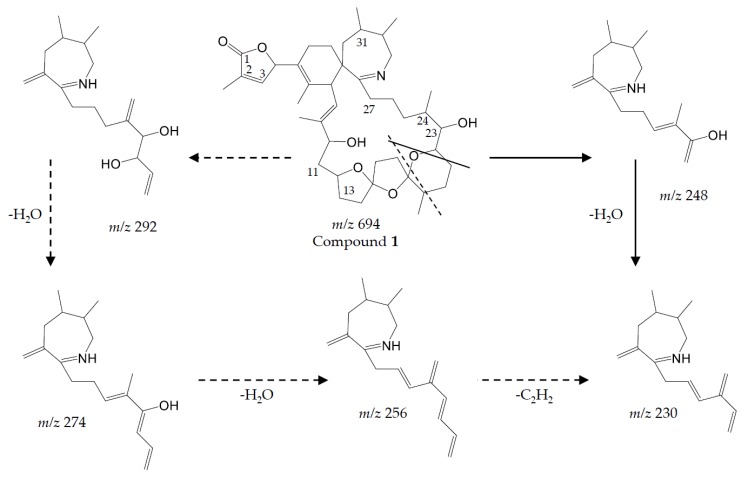
Putative fragmentation pattern of *m*/*z* 230–292 fragments of compound (**1**).

Compound (**1**) with a molecular formula C_41_H_60_O_8_N presents one methylene less and one oxygen more than 13-desMethyl-spirolide C (C_42_H_62_O_7_N). Its CID spectrum displays a molecular ion cluster that shows four subsequent water losses, whereas the CID spectrum of 13-desMethyl-spirolide C only shows three water losses (Figure 2A,B). This confirms the presence of an additional hydroxyl group in (**1**). Moreover, in contrast to 13-desMethyl-spirolide C, the A-type fragment cluster of (**1**) was down-shifted of 16 Da (Figure 2A,B), which clearly indicated that the additional hydroxyl group must be in the C1 to C11 part of the molecule (Figure 5). On the other hand, the spectrum of (**1**) showed an additional cluster including ions at *m/z* 230, 248, 256, 274 and 292 (Figure 2B) that is typically not observed in CID spectra of SPX. This unusual formation of the *m/z* 248 fragment can be explained by the typical SPX ether ring cleavage (solid line in Figure 5) including an additional hydroxyl group at the C22–C27 part of the structure. However, in contrast to 27-Hydroxy-13-desMethyl-spirolide C, (**1**) does not produce a *m/z* 180 fragment and 27-Hydroxy-13-desMethyl-spirolide C does not produce the above-mentioned atypical ion cluster observed in (**1**) (Figure 2D) indicating that the hydroxylation cannot be at the C27 position. On the other hand, the fragments *m/z* 292, 274 and 256 can be explained by a modified cleavage of the ether ring (dashed lines in Figure 5). Such a modification requires a different chemistry in close vicinity of the ether ring, which argues for a hydroxylation at C22 or C23. In summary, interpretation of the CID fragments versus those of 13-desMethyl-spirolide C indicates the presence of a hydroxyl group between C1 and C11 and another one most likely at C23. The structure of (**1**) would thus be consistent with the structure of 11,23-diHydroxy-19-deHydroxy-13,19-didesMethyl-spirolide C.

##### Compound **2**

The CID spectrum of (**2**) (Figure 2C) is identical to that of (**1**) (Figure 2B) except for a 2 Da up-shift (Table 5) of the molecular ion cluster. This indicates a reduction of a double bond in the part of the molecule between C1 and C12. In this part of the molecule, there are two double bonds that may be reduced: Δ^2,3^ and Δ^8,9^. Since a reduction of the double bond in the butenolide ring (C2/3) of the molecule has been observed in other SPX-like compounds such as spirolide B and D, which are 2,3-reduced forms of spirolides A and C [20], it is thus reasonable to assume that (**2**) is the Δ^2,3^ reduced form (instead of the Δ^8,9^) of compound (**1**). 

##### Compound **3**

Compound (**3**) displays an identical CID spectrum as 13-desMethyl-spirolide C (Figure 2E) except for the pseudo molecular ion cluster, which is up-shifted of 18 Da. This and the elemental formula of C_42_H_64_O_8_N (Table 5) indicate a reduction of a double bond and a hydroxylation in the C1 to C11 part of the molecule in comparison to 13-desMethyl-spirolide C (C_42_H_62_O_7_N), or even an opening of the lactone ring, which, however, has never been observed so far. However, due to lacking fragmentation of this part of the molecule, the positions of these modifications cannot be determined by mass spectral analyses.

##### Compound **4**

In contrast, the C1 to C11 part of (**4**) is identical with 13-desMethyl-spirolide C as mass differences from the pseudo molecular ions and the A-type fragments of both compounds are identical (230 Da; 692–462 and 720–490) (Figure 2A,F). The mass difference between (**4**) and 13-desMethyl-spirolide C is 28 Da and corresponds to two methylene groups, which, in comparison to 13-desMethyl-spirolide C, could be two additional methyl groups in the C12 to C33 part of the molecule, extensions of the carbon chain or a combination of both. Since no other fragments are formed, a more precise localization of these modifications is not possible by mass spectrometry alone.

##### Compound **5**

Compound (**5**) in turn shows an identical CID spectrum as (**4**), except for an up-shift of 2 Da of the pseudo molecular ion cluster (Figure 2F,G). Like in the case of (**1**) and (**2**) as discussed above, this mass shift most likely results from a reduction of one of the two double bonds (Δ^2,3^ or Δ^8,9^).

#### 4.2.2. Gymnodimines

In addition to the seven SPX, the Dutch strains produced at least four different GYM. Reports about the occurrence of both SPX and GYM in *A. ostenfeldii* are relatively recent, with the first record of 12-Me-GYM A being reported in *A. ostenfeldii* strains from the U.S. [26]. Gymnodimine A first has been isolated from shellfish harvested in New Zealand [31], and consequently from a toxic *Gymnodinium* sp. [50], but were first reported in *A. ostenfeldii* from The Netherlands [32], and preliminarily had been detected in Canadian *A. ostenfeldii* [51].

**Figure 6:**
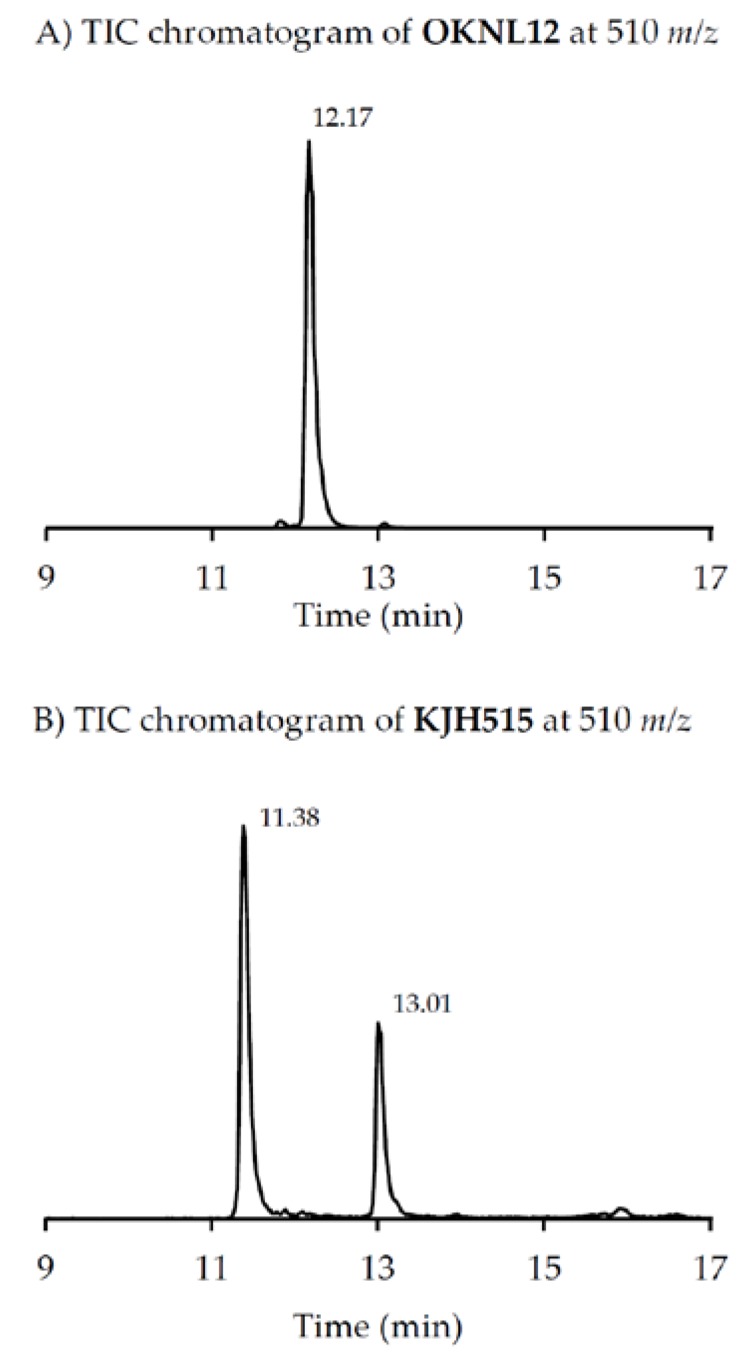
Total Ion Chromatograms (TIC) of *A. ostenfeldii* strains (**A**) OKNL12 and (**B**) KJH515.

Two of the gymnodimines of the Dutch *A. ostenfeldii* (GYM A and 12-Me-GYM A) have been described in the previous analysis of the same population [32]. However, our in-depth analysis revealed the presence of two additional gymnodimines so far unreported. While GYM A and 12-Me-GYM A were unambiguously identified by comparison to the reference standards, evidence for the classification of compounds (**6**) and (**7**) as GYM is that they elute within a small retention time window and that they fall in the same mass range. Most importantly, the CID spectra of GYM A and 12-Me-GYM A on the one hand, and those of (**6**) ([M + H]^+^ *m/z* 510) and (**7**) ([M + H]^+^ *m/z* 526) on the other hand, share common features. CID spectra of GYM are not very characteristic, but they show several low-mass fragment ion clusters at *m/z* 120, 136, 162, 174 and 202 with *m/z* 136 being the most abundant of the aforementioned ions (Figure 3A,C). These patterns are shared among all four spectra, which indicate a chemical similarity between all four compounds. Especially, (**6**) and (**7**) show almost identical spectra except for an up-shift of 16 Da of the fragments above *m/z* 300, indicating that (**7**) is a hydroxylated form of (**6**) (Figure 3B,D). Besides the new GYM D (*m/z* 524), very recently two other GYM analogues with *m/z* 510 (without structural elucidation) have been found in Baltic strains of *A. ostenfeldii* [33], but the compound of the Dutch strains is a third compound as it can be chromatographically separated from the other two analogues (Figure 6). Comparison of the mass spectrum of (**6**) with that of gymnodimine D [33] showed very close similarities. Interestingly, (**6**) (major compound) has a mass difference of 14 Da to gymnodimine D ([M+H]^+^ at *m/z* 524) (Figure 3B; [33]). Furthermore, intense product ions were detected at *m/z* 332 and 302 (Figure 2B), which correspond to the typical product ions at *m/z* 346 and 316 detected for gymnodimine D [33] also with a 14 Da mass difference. The difference between the compounds must be located between carbons 16–21 in gymnodimine D, and (**6**) apparently is a gymnodimine D analogue with one carbon shorter chain in the macrocyclic ring or a demethylated gymnodimine D.

### 4.3. Qualitative and Quantitative Variability among Strains

As discussed before, structural diversity of spiroimines within the population was high. Moreover, variability in spiroimine profiles among strains was high. Whereas some spiroimines (2 out of 11, namely 13-desMeC SPX and compound **6**) were present in all strains, the majority (9 out of 11) was lacking in a few strains (Appendix A, Table A4). The most plausible reason for the presence of different spiroimines in different *A. ostenfeldii* strains is a differing presence and/or expression of biosynthetic genes in these strains, but the possibility that some low-expressed spiroimines were below the detection limit in some strains cannot be ruled out (Appendix A, Table A1 and Table A2).

Notably, the spiroimines which were detected in most strains include both quantitatively dominant compounds (e.g., **6**) and minor compounds (e.g., **3**). On the other hand, congeners not detectable in all strains include compounds generally low in quantity (e.g., **5**) or compounds (e.g., **2**) that were found in just a few strains, and at times being a dominant compound (Table 4, Figure 4).

Most importantly, a new gymnodimine that was the most abundant spiroimine was detected in the Dutch *A. ostenfeldii*. This major GYM (**6**) previously had been overlooked due to the targeted approach of the Selected Reaction Monitoring (SRM) that only detects compounds that a priori are included in the analyte list. However, the detection of GYM D and numerous related compounds [33] with a more sensitive Orbitrap mass spectrometer in the full scan mode prompted us to search for these compounds as well. Under the assumption of an identical molar response of (**6**) and GYM A, the maximum cell quota of (**6**) reached up to 274 pg cell^−1^ (GYM A equivalents), which corresponds to a 10 to 1000-fold higher cell quota of the various strains than the two gymnodimines reported before. This convincingly shows that qualitative and quantitative comparisons of spiroimines of different *A. ostenfeldii* strains depend on the analytical depth with which samples are analysed and the compatibility of the applied methods.

A high diversity and large intraspecific variability of cell quotas suggests that none of the spiroimine compounds has a vital role in primary cell metabolism, but that they are secondary metabolites. It is difficult to determine the evolutionary drivers for such a high diversity and variability of spiroimines as their ecological function remains unknown. It has been suggested that gymnodimines and spirolide share a common biosynthetic pathway [26], which may be an indication for a common function of both toxin classes. The toxic effect on vertebrates has been shown to be dependent of the cycloimine moiety of the molecules [21]. When the cycloimine function is cleaved by hydrolysis, the compounds lose their toxicity. However, the cycloimine moiety apparently is not the only pre-requisite for toxicity: It has been shown [25] that spirolides H and I were non-toxic, even though they possessed the cycloimine moiety. However, these two spirolides consisted only of a dispiroketal ring system in the macrocycle instead of a trispiroketal system as in all the other spirolides. This is clear evidence that, in the case of spirolides, toxicity does not depend on a single structural element alone. On the other hand, it should be considered that vertebrate toxicity most likely is not the true ecological function of spiroimines but rather is a coincidental side effect. The presence of high amounts of complex molecules requiring elaborate synthesis machinery implies that there should be a role or benefit for the producing cell. However, clear evidence for such a role of spiroimines, e.g., in interactions with potential grazers or competitors, is lacking.

Whatever the role and function of spiroimines is, structural variability does not seem to fundamentally influence it; otherwise, one would expect more conserved structures. High structural variability is certainly not an exclusive feature of spiroimines. For example, 93 structural yessotoxin variants were found in one strain of *Protoceratium reticulatum* [52], and numerous analogues of azaspiracids have been detected in different strains of Amphidomataceae [53]. Modifications of the, in most cases conserved, polyketide backbone include methylations and demethylations, hydration of double bonds or additional unsaturations, hydroxylations and dehydroxylations, to name the most common ones. In some cases, chain extensions or shortenings have also been observed. In a few cases, glycosylations [52] and phosphorylations [54] were reported. 

As a general conclusion, our results, together with other recent reports on *A. ostenfeldii* [11,33], point out that spiroimines diversity in *A. ostenfeldii* probably is underestimated and deserves increased attention, and that, for comparison, *A. ostenfeldii* strains from other locations should be analysed with comparable methods and analytical depth. For the new and abundant spiroimine congeners, information on their toxicity is needed to evaluate their potential risk for human health. Finally, our multi-strain study revealed that studying one or a very few strains of a microalgal population is of limited suitability to fully describe secondary metabolite diversity and variability.

## Figures and Tables

**Table 1:** Structures of known spirolides [24,25,26,34] and corresponding *m/z* of their [M + H]^+^ ions.

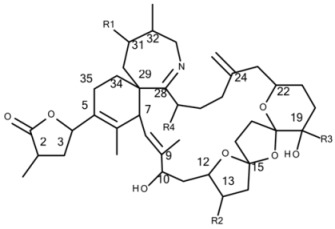							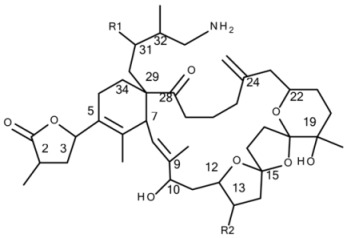				
**Spirolide**	**R1**	**R2**	**R3**	**R4**	**∆^2,3^**	***m/z***	**Spirolide**	**R1**	**R2**	**∆^2,3^**	***m/z***
A	H	CH_3_	CH_3_	H	+	692.5	E	H	CH_3_	+	710.5
B	H	CH_3_	CH_3_	H	−	694.5	F	H	CH_3_	−	712.5
C	CH_3_	CH_3_	CH_3_	H	+	706.5					
D	CH_3_	CH_3_	CH_3_	H	−	708.5					
13-desMe C	CH_3_	H	CH_3_	H	+	692.5					
13,19-didesMe C	CH_3_	H	H	H	+	678.5					
27-Hydroxy-13-desMe C	CH_3_	H	CH_3_	OH	+	694.5					
27-oxo-13-desMe C	CH_3_	H	CH_3_	=O	+	692.5					
13-desMe D	CH_3_	H	CH_3_	H	−	694.5					
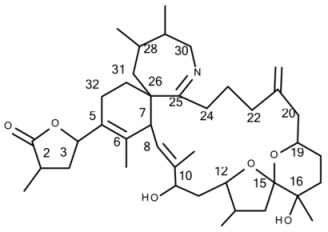							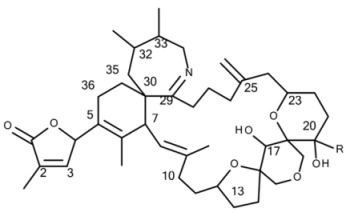				
**Spirolide**	**∆^2,3^**			***m/z***			**Spirolide**	**R**		***m/z***	
H	+			650.5			G	H		692.5	
I	−			652.5			20-Me G	CH_3_		706.5	

**Table 2:** Mass transitions of spiroimines included in liquid chromatography-tandem mass spectrometry (LC-MS/MS) analysis (*m/z*; precursor ion → fragment ion), retention times and characteristic group fragments. Compounds detected in *A. ostenfeldii* from The Netherlands and their respective data are printed in bold.

Mass Transition (*m/z*)	Common Name	Reference	Retention Time (min)	*m/z*			
				Group 1	Group 2	Group 3	Group 4
Spirolides							
650 → 164	Spirolide H	[25]		650/632/614	402/384	206	164
652 → 164	Spirolide I	[25]		652/634/616	402/384	206	164
678 → 164	13,19-didesMethyl-spirolide C	[23,36]		678/660/642/624	448/430/412/394		164
692 → 150	Spirolide A	[37]		692/674/624	444/390	190	150
**692 → 164**	**13-desMethyl-spirolide C**	**This study, [37]**	**12.76**	**692/674/656/638**	**462/444/426**		**164**
692 → 164	Spirolide G	[22]		692/674/656/638	378		164
694 → 150	Spirolide B	[20,21]		694/676/658/640	462/444/426		150
694 → 164	13-desMethyl spirolide D	[38]		694/676/658/640	444/426	230/204/177	164
**694 → 164**	**(1)**	**This study**	**12.33**	**694/676/658/640/622**	**446/428/410**	**292/274/248**	**164**
**696 → 164**	**(2)**	**This study**	**12.39**	**696/678/660/642/624**	**464/446/428/410**	**292/274/248**	**164**
706 → 164	Spirolide C	[37]		706/688/638	458/404	204	164
706 → 164	20-Methyl-spirolide G	[39]		706/688/670/652	392/374/346	258	164
708 → 164	Spirolide D	[20,39]		708/690/672/654	458/440	230/206/204/177	164
**708 → 180**	**27-Hydroxy-13-desMethyl-spirolide C**	**This study, [24]**	**13.06**	**708/690/672/654/636**	**478/460/442/424**		**180**
**710 → 164**	**(3)**	**This study**	**12.97**	**710/692/674/656/638**	**462/444/426**		**164**
**720 → 164**	**(4)**	**This study**	**13.29**	**720/702/684/666**	**490/472/454**		**164**
**722 → 164**	**(5)**	**This study**	**13.40**	**722/704/686/668**	**490/472/454**		**164**
Gymnodimines							
**508 → 490**	**Gymnodimine A**	**This study, [31]**	**11.81**	**508/490/392/286/246/202/174/162/136/121**			
**510 → 492**	**(6)**	**This study**	**12.21**	**510/492/482/332/302/136/120**			
**522 → 504**	**12-Methyl-gymnodimine A**	**This study, [26]**	**12.17**	**522/504/406/300/246/202/174/162/136/120**			
**526 → 508**	**(7)**	**This study**	**11.32**	**526/508/348/262/174/162/136/120**			

**Table 3:** Calculated and measured accurate masses (*m/z*) for [M + H]^+^ at *m/z* 510 and its product ions obtained with Liquid Chromatography–High-Resolution Mass Spectrometry (LC–HRMS).

Formula	Relative Intensity (%)	Calculated	Measured	Δ, ppm
C_31_H_44_O_5_N^+^	100	510.3214	510.3224	2.0
C_31_H_42_O_4_N^+^	62	492.3108	492.3119	2.1
C_30_H_44_O_4_N^+^	14	482.3265	482.3278	2.7
C_31_H_40_O_3_N^+^	6	474.3003	474.3011	1.7
C_30_H_44_O_3_N^+^	7	466.3316	466.3325	2.0
C_30_H_42_O_3_N^+^	5	464.3159	464.3167	1.6
C_29_H_38_O_4_N^+^	3	464.2795	464.2805	2.0
C_30_H_42_O_2_N^+^	7	448.3210	448.3218	1.8
C_23_H_34_O_4_N^+^	7	388.2482	388.2489	1.7
C_20_H_30_O_3_N^+^	29	332.2220	332.2225	1.6
C_20_H_28_O_2_N^+^	3	314.2115	314.2119	1.5
C_19_H_28_O_2_N^+^	6	302.2115	302.2119	1.6
C_17_H_26_ON^+^	3	260.2009	260.2013	1.4
C_17_H_24_ON^+^	2	258.1852	258.1857	1.8
C_14_H_20_N^+^	4	202.1590	202.1595	2.1
C_13_H_20_N^+^	5	190.1590	190.1594	2.0
C_13_H_18_N^+^	3	188.1434	188.1438	2.2
C_11_H_16_N^+^	3	162.1277	162.1281	2.3
C_11_H_14_N^+^	2	160.1121	160.1125	2.3

**Table 4:** Range and fold change of cell quota of PST and spiroimine compounds. Transitions are mentioned in *m/z*. Samples size was *N* = 68; n_i_ describes the absolute number of strains in which the toxin was detected. Numbers in brackets next to “<LOD” indicate the lowest level of compound recorded above detection limit which was used to calculate fold-changes (LOD = Limit of detection; PST = paralytic shellfish toxins; STX = saxitoxin; GTX = gonyautoxins; GYM = gymnodimines).

Transition	Compound	n_i_	Minimum (fg cell^−1^)	Maximum (fg cell^−1^)	Fold Change
PST					
	STX	68	370	6800	18
	GTX2/3	68	1500	12,800	8
	B1	67	<LOD (120)	940	9
	C1/C2	68	7200	74,400	10
Spirolides					
692-164	13-desMe C	68	20	5500	273
694-164	(**1**)	7	<LOD (20)	970	42
696-164	(**2**)	2	<LOD (920)	1100	1
708-180	27-Hydroxy-13-desMe C	37	<LOD (10)	80	6
710-164	(**3**)	54	<LOD (10)	60	5
720-164	(**4**)	45	<LOD (30)	280	8
722-164	(**5**)	47	<LOD (10)	140	10
Gymnodimines					
508-490	GYM A	58	<LOD (310)	18,100	59
510-492	(**6**)	68	37,000	274,000	7
522-504	12-Me-GYM A	30	<LOD (300)	1500	5
526-508	(**7**)	63	<LOD (530)	26,500	50

**Table 5:** Exact masses of the pseudo-molecular ions and elemental formula of spirolides contained in *A. ostenfeldii* extract measured on the LTQ-Orbitrap MS. Ring double bond (RDB) equivalents and errors in ion assignments (ppm) are also reported.

Name	[M + H]^+^, *m/z*	Formula	RDB	Δ, ppm
Spirolides				
13-desMe C	692.4521	C_42_H_62_O_7_N	12.5	0.029
(**1**)	694.4322	C_41_H_60_O_8_N	12.5	1.232
(**2**)	696.4474	C_41_H_62_O_8_N	11.5	0.583
27-Hydroxy-13-desMe C	708.4477	C_42_H_62_O_8_N	11.5	0.996
(**3**)	710.4615	C_42_H_64_O_8_N	11.5	−1.611
(**4**)	720.4817	C_44_H_66_O_7_N	12.5	2.331
(**5**)	722.4974	C_44_H_68_O_7_N	11.5	−2.256
Gymnodimines				
GYM A	508.3417	C_32_H_46_O_4_N	10.5	−0.856
(**6**)	510.3208	C_31_H_44_O_5_N	10.5	−1.176
12-Me-GYM A	522.3575	C_33_H_48_O_4_N	10.5	−0.546
(**7**)	526.3158	C_31_H_44_O_6_N	10.5	−0.978

## Appendix A

**Table A1:** Mean limits of detection (LOD, S/N = 3) for PST based on mean cell numbers of the analyzed strains. Values are expressed in fg cell^−1^.

Standard	LOD
C1/2	5.1
GTX 2	2.2
B1	9.4
GTX 3	2.5
STX	2.1

**Table A2:** Mean limits of detection (LOD, S/N = 3) for spiroimines based on mean cell numbers of the analyzed strains. Values are expressed in fg cell^−1^.

Standard	LOD
13-desMe C	13.7
GYM A	290.3

**Table A3:** Cell quotas of PST. Values are expressed in pg cell^−1^.

Strain	C1/C2	B1	GTX2/3	STX	Total
OKNL-11	40.88	0.79	5.63	6.81	54.11
OKNL-12	30.93	0.19	4.46	0.71	36.29
OKNL-13	31.89	0.31	5.35	1.14	38.69
OKNL-14	54.90	0.41	7.98	1.06	64.35
OKNL-15	44.19	0.33	5.79	2.00	52.31
OKNL-16	19.50	0.26	2.41	3.41	25.58
OKNL-17	42.31	0.36	4.02	1.04	47.73
OKNL-18	30.81	0.21	5.31	0.76	37.09
OKNL-19	69.14	0.48	9.65	2.50	81.77
OKNL-20	49.66	0.23	5.15	1.44	56.48
OKNL-21	60.81	0.42	8.06	2.23	71.52
OKNL-22	66.58	0.41	12.66	1.91	81.56
OKNL-23	27.59	0.24	3.79	0.98	32.60
OKNL-24	35.42	0.33	3.58	1.77	41.10
OKNL-25	47.45	<LOD	7.90	0.91	56.26
OKNL-26	38.41	0.32	6.98	1.36	47.07
OKNL-27	67.42	0.39	10.67	2.73	81.21
OKNL-28	66.96	0.56	6.13	2.27	75.92
OKNL-29	29.69	0.36	7.78	1.91	39.74
OKNL-30	49.23	0.68	5.62	4.40	59.93
OKNL-31	24.07	0.40	2.78	2.19	29.44
OKNL-32	40.88	0.47	6.78	1.34	49.47
OKNL-33	44.76	0.59	5.25	2.13	52.73
OKNL-34	18.45	0.26	2.79	0.88	22.38
OKNL-35	45.52	0.67	4.45	2.23	52.87
OKNL-36	32.81	0.24	4.71	1.31	39.07
OKNL-37	36.84	0.25	4.00	0.51	41.60
OKNL-38	27.96	0.28	4.90	1.47	34.61
OKNL-39	33.09	0.22	4.85	0.68	38.84
OKNL-40	45.90	0.22	6.83	2.07	55.02
OKNL-41	15.19	0.23	1.62	0.82	17.86
OKNL-42	34.36	0.27	4.44	1.38	40.45
OKNL-43	50.54	0.68	5.89	3.06	60.17
OKNL-44	35.87	0.33	5.02	1.89	43.11
OKNL-45	25.69	0.28	3.58	1.02	30.57
OKNL-46	32.84	0.22	5.90	1.12	40.08
OKNL-47	46.64	0.68	5.85	3.41	56.58
OKNL-48	36.29	0.51	4.92	1.44	43.16
OKNL-49	39.51	0.54	5.78	1.70	47.53
OKNL-50	53.19	0.94	6.14	1.63	61.90
OKNL-51	41.87	0.88	6.80	2.21	51.76
OKNL-52	32.79	0.56	4.12	1.27	38.74
OKNL-53	74.36	0.54	11.72	1.53	88.15
OKNL-54	32.96	0.60	7.18	1.18	41.92
OKNL-55	32.09	0.49	3.60	2.53	38.71
OKNL-56	53.76	0.83	12.35	2.13	69.07
OKNL-57	13.63	0.54	2.70	0.67	17.54
OKNL-58	43.63	0.50	7.93	0.91	52.97
OKNL-59	24.79	0.51	3.61	2.14	31.05
OKNL-60	22.33	0.41	3.97	1.32	28.03
OKNL-61	29.57	0.47	4.29	1.21	35.54
OKNL-62	19.53	0.19	3.18	0.52	23.42
OKNL-63	43.44	0.69	6.01	0.48	50.62
OKNL-64	57.14	0.80	12.78	2.31	73.03
OKNL-65	59.24	0.77	9.03	2.93	71.97
OKNL-66	24.36	0.45	4.84	1.23	30.88
OKNL-67	44.26	0.63	7.63	1.01	53.53
OKNL-68	7.20	0.50	6.79	1.65	16.14
OKNL-69	46.47	0.71	10.85	1.03	59.06
OKNL-70	30.94	0.39	5.15	1.13	37.61
OKNL-71	59.85	0.70	11.35	3.12	75.02
OKNL-72	23.02	0.49	3.50	0.52	27.53
OKNL-73	18.45	0.60	3.05	2.56	24.66
OKNL-74	9.00	0.22	1.76	0.37	11.35
OKNL-75	30.15	0.19	2.67	0.56	33.57
OKNL-76	32.89	0.33	3.85	0.92	37.99
OKNL-77	12.50	0.12	1.51	0.48	14.61
OKNL-78	29.99	0.24	4.32	0.72	35.27

**Table A4:** Cell quotas of spiroimines. Values are expressed in fg cell^−1^.

Strain	GYM A	12-Me-GYM A	13-desMe C	(1)	(2)	27-Hydroxy-13-desMe C	(3)	(4)	(5)	(6)	(7)	Total
OKNL-11	928	<LOD	343	<LOD	<LOD	28	18	152	62	200,617	4085	206,233
OKNL-12	7282	1548	5463	<LOD	<LOD	76	55	<LOD	<LOD	197,589	26,529	238,542
OKNL-13	<LOD	<LOD	303	<LOD	<LOD	<LOD	<LOD	<LOD	<LOD	121,491	<LOD	121,794
OKNL-14	3395	1073	408	<LOD	<LOD	24	36	123	63	150,060	7675	162,857
OKNL-15	16,658	<LOD	1586	<LOD	<LOD	42	35	<LOD	<LOD	223,220	<LOD	241,541
OKNL-16	1477	361	255	<LOD	<LOD	<LOD	16	58	31	115,886	2421	120,505
OKNL-17	10,047	<LOD	280	<LOD	<LOD	<LOD	27	<LOD	27	197,291	4821	212,493
OKNL-18	4041	603	392	<LOD	<LOD	18	28	<LOD	48	194,480	2935	202,545
OKNL-19	4518	1235	101	25	<LOD	<LOD	16	<LOD	<LOD	176,732	3785	186,412
OKNL-20	15,520	<LOD	314	<LOD	<LOD	34	37	<LOD	<LOD	268,805	10,896	295,606
OKNL-21	1907	431	94	23	<LOD	<LOD	<LOD	<LOD	<LOD	86,686	2288	91,429
OKNL-22	12,383	<LOD	302	<LOD	<LOD	16	18	139	58	199,729	1245	213,890
OKNL-23	5901	<LOD	213	<LOD	<LOD	<LOD	14	83	40	63,366	13,079	82,696
OKNL-24	<LOD	<LOD	157	<LOD	<LOD	<LOD	15	<LOD	<LOD	150,658	2455	153,285
OKNL-25	1474	<LOD	312	<LOD	<LOD	<LOD	25	61	23	118,422	2125	122,442
OKNL-26	1203	<LOD	20	<LOD	<LOD	<LOD	<LOD	52	31	140,922	2672	144,900
OKNL-27	<LOD	<LOD	1401	<LOD	<LOD	25	22	139	53	208,334	528	210,502
OKNL-28	<LOD	<LOD	149	<LOD	<LOD	22	14	146	55	274,020	788	275,194
OKNL-29	1005	319	114	<LOD	<LOD	<LOD	14	41	16	74,436	1813	77,758
OKNL-30	11,024	<LOD	75	<LOD	<LOD	<LOD	<LOD	<LOD	<LOD	121,534	<LOD	132,633
OKNL-31	986	<LOD	521	<LOD	<LOD	<LOD	<LOD	53	19	73,498	1473	76,550
OKNL-32	943	<LOD	459	<LOD	<LOD	<LOD	21	45	19	86,256	1479	89,222
OKNL-33	4641	898	192	<LOD	<LOD	18	<LOD	80	32	121,749	2230	129,840
OKNL-34	1721	579	620	<LOD	<LOD	<LOD	23	69	35	98,643	1907	103,597
OKNL-35	308	<LOD	2124	470	921	55	60	191	74	261,723	5365	271,291
OKNL-36	1351	<LOD	452	<LOD	<LOD	<LOD	15	76	37	122,714	1553	126,198
OKNL-37	4374	857	681	<LOD	<LOD	14	16	107	53	75,678	2295	84,075
OKNL-38	1368	435	1954	<LOD	<LOD	<LOD	18	81	39	93,132	1817	98,844
OKNL-39	4874	<LOD	448	<LOD	<LOD	<LOD	16	<LOD	<LOD	93,171	<LOD	98,509
OKNL-40	<LOD	<LOD	1184	<LOD	<LOD	15	26	182	73	215,406	2539	219,425
OKNL-41	1606	356	300	<LOD	<LOD	<LOD	14	56	26	94,071	1836	98,265
OKNL-42	4863	1110	2921	<LOD	<LOD	49	58	276	143	226,343	9711	245,474
OKNL-43	18,125	<LOD	502	<LOD	<LOD	28	27	266	105	232,667	1772	253,492
OKNL-44	12,242	<LOD	356	<LOD	<LOD	27	38	192	77	163,653	3179	179,764
OKNL-45	3783	803	2496	<LOD	<LOD	26	47	<LOD	<LOD	139,828	1668	148,651
OKNL-46	7499	<LOD	700	<LOD	<LOD	<LOD	<LOD	<LOD	<LOD	76,279	2480	86,958
OKNL-47	6233	1263	2026	<LOD	<LOD	43	19	<LOD	<LOD	134,643	5576	149,803
OKNL-48	9668	<LOD	1387	609	1136	21	17	<LOD	<LOD	104,590	3871	121,299
OKNL-49	16,028	<LOD	709	<LOD	<LOD	18	21	264	98	202,718	1393	221,249
OKNL-50	1053	<LOD	930	285	<LOD	21	53	208	88	197,493	6347	206,478
OKNL-51	5840	<LOD	287	<LOD	<LOD	<LOD	<LOD	66	23	98,564	948	105,728
OKNL-52	3384	588	857	<LOD	<LOD	17	38	120	51	117,763	4273	127,091
OKNL-53	5845	879	1622	<LOD	<LOD	42	50	216	74	190,846	2657	202,231
OKNL-54	1627	485	694	<LOD	<LOD	<LOD	23	56	29	93,308	<LOD	96,222
OKNL-55	12,160	<LOD	2041	<LOD	<LOD	18	28	156	67	114,101	5323	133,894
OKNL-56	14,775	<LOD	996	<LOD	<LOD	22	48	132	44	127,744	5740	149,501
OKNL-57	1267	343	802	<LOD	<LOD	<LOD	17	<LOD	<LOD	53,936	1523	57,888
OKNL-58	3637	1223	2789	<LOD	<LOD	37	54	206	107	159,495	5243	172,791
OKNL-59	3340	710	2774	<LOD	<LOD	26	22	<LOD	<LOD	120,110	3369	130,351
OKNL-60	2020	436	977	<LOD	<LOD	<LOD	19	<LOD	<LOD	73,686	1974	79,112
OKNL-61	2213	608	988	<LOD	<LOD	19	<LOD	<LOD	<LOD	91,097	2484	97,409
OKNL-62	2899	<LOD	593	<LOD	<LOD	<LOD	<LOD	44	23	61,320	1730	66,609
OKNL-63	2588	725	215	<LOD	<LOD	<LOD	20	<LOD	<LOD	114,407	943	118,898
OKNL-64	<LOD	<LOD	2083	<LOD	<LOD	22	36	194	73	180,477	7084	189,969
OKNL-65	<LOD	<LOD	280	<LOD	<LOD	<LOD	37	108	44	162,664	3674	166,807
OKNL-66	3875	<LOD	326	<LOD	<LOD	<LOD	<LOD	47	21	65,556	1798	71,623
OKNL-67	<LOD	<LOD	1012	<LOD	<LOD	17	21	117	40	149,944	3865	155,016
OKNL-68	6673	<LOD	2775	968	<LOD	19	34	<LOD	<LOD	117,031	1174	128,674
OKNL-69	5131	728	158	<LOD	<LOD	18	23	<LOD	<LOD	175,549	6465	188,072
OKNL-70	3062	683	2940	567	<LOD	23	35	148	66	128,484	4025	140,033
OKNL-71	<LOD	295	1102	<LOD	<LOD	24	56	134	46	198,947	1074	201,678
OKNL-72	1114	337	606	<LOD	<LOD	<LOD	14	46	23	47,892	953	50,985
OKNL-73	7290	<LOD	1486	<LOD	<LOD	25	27	95	34	82,527	2609	94,093
OKNL-74	2771	<LOD	265	<LOD	<LOD	<LOD	<LOD	56	27	36,980	1009	41,108
OKNL-75	2878	593	425	<LOD	<LOD	16	20	<LOD	<LOD	128,412	2731	135,075
OKNL-76	<LOD	<LOD	487	<LOD	<LOD	14	14	102	43	158,303	4002	162,965
OKNL-77	2195	397	416	<LOD	<LOD	<LOD	<LOD	34	15	45,188	1068	49,313
OKNL-78	7656	<LOD	82	<LOD	<LOD	<LOD	<LOD	59	27	87,556	2968	98,348
